# Supplementary material for: Correlating Stability-Indicating Biochemical and Biophysical Characteristics with In Vitro Cell Potency in mRNA LNP Vaccine
Source: Vaccines (Basel). 2024 Feb 7;12(2):169. doi: 10.3390/vaccines12020169 (PMC10893231; doi:10.3390/vaccines12020169)
Supplement: Supplementary file 1 [file vaccines-12-00169-s001.zip › vaccines-2839530-supplementary.pdf]

Supplemental Materials for

Correlating Stability-Indicating Biochemical and Biophysical Characteristics with In Vitro Cell Potency in mRNA LNP Vaccine

1. Study design for the short-term stability study

To study the correlation between lipid nanoparticle (LNP) physiochemical properties and *in vitro* potency, a short-term (three-month) stability study was designed. Based on preliminary studies, we identified that the *in vitro* potency of LNPs would considerably decrease after three months. The mRNA LNP materials were developed and manufactured in-house. After manufacturing, the mRNA LNP samples were stored at -70°C and tested as the Time 0 results. The mRNA LNP samples were then placed in conditions at -70°C, -20°C, 4°C, 25°C, 37°C, and 45°C for three months. Aliquots were tested for mRNA integrity, LNP particle size, encapsulation efficiency, and *in vitro* potency at the corresponsive time points (Table S1).

Table S1. A summarized study design table for the short-term (three-month) stability study.

**Condition**	**Time Point**								
	T=0	1 Day	2 Days	4 Days	8 Days	16 Days	1 Month	2 Months	3 Months
**45˚C**		X	X	X	X				
**37˚C**		X	X	X	X	X	X		
**25˚C**			X		X	X	X	X	X
**4˚C**						X	X	X	X
**-20˚C**							X	X	X
**-70˚C**	X								

* X = sample taken.

2. Results of the short-term stability study

2.1. The stability of LNP's physiochemical properties

Important physiochemical characterizations, such as LNP particle size (Figure S1 (A)) and encapsulation efficiency (Figure S1 (B)), were monitored at various time points.

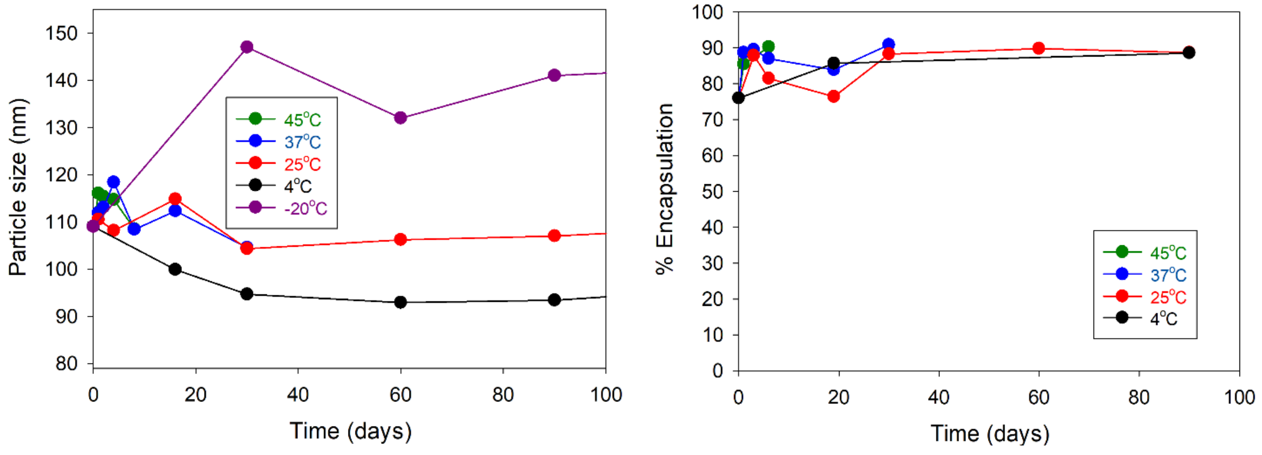

(A) (B)

Figure S1. LNP physiochemical properties change over time. Results (y-axis) from (A) particle size, and (B) encapsulation efficiency were plotted against time (days, x-axis). Each data point represents the average result tested at a single time point within their respective storage condition. For particle size testing, samples were tested in duplicate (N=2). For encapsulation efficiency testing, samples were tested in quadruplicate (N=4).

For mRNA LNP samples stored at 4°C, 25°C, 37°C, and 45°C, particle sizes tested by DLS all fit within the range of 90 nm to 120 nm. For samples stored at -20°C, the LNP size tested remained large (above 130 nm) through the 3-month time points. On the contrary, no significant differences existed in the encapsulation efficiency tested for mRNA LNP samples. Even under elevated conditions, the majority of tested encapsulation efficiency (EE) remained close to 90%. The results were aligned with our historical data (unpublished data). Previously, mRNA LNP samples were stored at -20 °C or -70 °C, including assay controls for the EE assay, for six months. We have seen no change in the %EE values regardless of the storage condition throughout the period of time. Therefore, we omitted the -20 °C condition for this short-term stability study.
